# Data in support of comparative analysis of strawberry proteome in response to controlled atmosphere and low temperature storage using a label-free quantification

**DOI:** 10.1016/j.dib.2015.02.023

**Published:** 2015-03-20

**Authors:** Li Li, Zisheng Luo, Xinhong Huang, Lu Zhang, Pengyu Zhao, Hongyuan Ma, Xihong Li, Zhaojun Ban, Xia Liu

**Affiliations:** a Key Laboratory of Food Nutrition and Safety (Ministry of Education), Tianjin University of Science and Technology, Tianjin 300457, PR China; b Department of Food Science and Nutrition, Zhejiang University, Hangzhou, Zhejiang 310058, PR China; c Jinan Fruit Research Institute, All China Federation of Supply and Marketing Cooperatives, Jinan, Shandong 250014, PR China; d College of Forestry and Horticulture, Xinjiang Agricultural University, Urumqi, Xinjiang 830052, PR China

## Abstract

To elucidate the mechanisms contributing to fruit responses to senescence and stressful environmental stimuli under low temperature (LT) and controlled atmosphere (CA) storage, a label-free quantitative proteomic investigation was conducted in strawberry (*Fragaria ananassa*, Duch. cv. ‘Akihime’). Postharvest volatile compounds were characterized following storage under different conditions. The observed post-storage protein expression profiles may be associated with delayed senescence features in strawberry [2]. A total of 454 proteins were identified in differentially treated strawberry fruits. Quantitative analysis, using normalized spectral counts, revealed 73 proteins common to all treatments, which formed three clusters in a hierarchical clustering analysis.

## Specifications table

**Table:** 

Subject area	*Biology*
More specific subject area	*Fruit proteomics*
Type of data	*Table, figure*
How data was acquired	• *Shimadzu QP2010 gas chromatograph–mass spectrometer (GC–MS) (Shimadzu Co., Kyoto, Japan)* • *LTQ XL mass spectrometer (Thermo Fisher) with a Michrom captive spray nano-electrospray ionization (NSI) source, low energy collision-induced dissociation (CID)* • *NCBI Viridiplantae entries, a total of 278115 sequences updated on Dec. 31th 2011 (NIH, Bethesda, MD, USA)*
Data format	*Analyzed*
Experimental factors	*Harvested strawberry (Fragaria ananassa, Duch. cv. ‘Akihime’) was transferred to the laboratory, sorted to discard damaged and diseased fruits, then calyxes and pedicels were removed. The surface of the strawberries was cleaned with a 2% sodium dodecyl sulfate (SDS) solution, before sorting strawberries into different storage treatments: either in package with controlled atmosphere comprised of 2% O*_*2*_*and 12% CO*_*2*_*, or in air at low temperature, or in air at room temperature. Samples for analysis were immediately frozen in liquid nitrogen, placed in sealable bags and stored at −80 °C. Three independent biological replicates were prepared using pooled tissue from twenty individual strawberries.*
Experimental features	• *Solid-phase microextraction (SPME) of volatile compounds* • *Label-free proteomic quantification*
Data source location	*Tianjin, China*
Data accessibility	*Data are available with this article.*

## Value of the data

• Label-free approach on analysis of the strawberry proteome in response to storage conditions.
• Coordinated changes in postharvest volatile evolution are characterized.
• Candidate proteins shown here that represent important metabolic pathways may contribute to storage tolerance were identified.

## 1 Data, experimental design, materials and methods

A total of 454 proteins were identified in differentially treated strawberry fruits. Quantitative analysis, using normalized spectral counts, revealed 73 proteins common to all treatments, which formed three clusters in a hierarchical clustering analysis.

### 1.1 Plant materials and treatments

Strawberries (*Fragaria ananassa*, Duch. cv. ‘Akihime’) were harvested from the Xintai Farmhouse, Tianjin Economical and Developmental Area, P.R. China. Harvested strawberry fruits were transferred to the laboratory, sorted to discard damaged and diseased fruits, then calyxes and pedicels were removed. The surface of the strawberries was cleaned with a 2% sodium dodecyl sulfate (SDS) solution, before sorting strawberries into different storage treatments: either in package with CA comprised of 2% O_2_ and 12% CO_2_, or in air at LT, or in air at RT. Sample fruits for analysis were immediately frozen in liquid nitrogen, placed in sealable bags and stored at −80 °C. Three independent biological replicates were prepared using pooled tissue from twenty individual strawberries.

### 1.2 Volatile determination

The volatile compounds of strawberry fruits were analyzed according to the protocol described by Li et al. [1]. The frozen fruits were first ground to a fine powder in liquid nitrogen using a stainless steel blender followed by a mortar and pestle. The weighed samples were homogenized with saturated NaCl solution and sealed hermetically in a sample vial for analysis. Volatiles were extracted using a solid-phase microextraction (SPME) fiber (DVB/Car/PDMS, Supleco Inc., Bellefonte, USA) coated with 100 μM polydimethylsiloxane (Supelco Inc., Bellefonte, USA) and inserted into the vial headspace, with an internal standard of 2-octanone in each sample. The sample vial was then heated at 60 °C for 1 h to facilitate the release of volatile compounds from the sample to the headspace.

The volatile compounds were separated on a DB-5MS column (J & W Scientific Inc., Folsom, USA) (30 m×0.25 mm i.d. ×0.25 µm film thickness) equipped with a Shimadzu QP2010 gas chromatograph–mass spectrometer (GC–MS) (Shimadzu Co., Kyoto, Japan). The carrier gas was ultra-pure helium (99.99%). The initial temperature of the column was 35 °C, which increased to a final temperature of 240 °C at the rate of 15 °C min^–1^, and then held at 240 °C for 4.5 min. Volatile components were identified by comparison of the collected mass spectra with reference spectra in a mass spectral library (National Institute of Standards and Technology, NIST). About 39 major volatile compounds were identified in all treatments after storage (Supplemental Table 1) and the complete linkage hierarchical clustering (Euclidean distance) performed using Pearson distances and Ward׳s algorithm for all compounds data aggravation are shown in Fig. 3 in Ref. [2].

### 1.3 LC–MS/MS analysis

Protein was extracted and purified from frozen, ground strawberry samples using phenol extraction followed by ammonium acetate–methanol precipitation [2]. Afterwards, the strawberry proteins were digested by trypsin in solution [2]. Digested peptides were desalted and separated with reversed-phase chromatography using a nano-HPLC system with a Capillary C18 5 μm particle, 150 μm×10 mm column (CTICAP5150100, Column Technology Inc). A binary solvent gradient was employed: solution A was composed of 0.1% formic acid and solution B composed of 100% acetonitrile and 0.1% formic acid. The 120 min gradient consisted of the following steps: 5% solution B for 15 min, from 5% to 32% solution B over 45 min, then increasing to 90% solution B in 35 min, decreasing from 90% to 5% solution B in 5 min, and then holding for 20 min. Separated peptides were analyzed in a LTQ XL mass spectrometer (Thermo Fisher) with a Michrom captive spray nano-electrospray ionization (NSI) source at a flow rate of 2 μL min^–1^. MS and MS/MS spectra were acquired, where the ten most abundant ions in the MS scan were selected for automated low energy collision-induced dissociation (CID); a 30 s exclusion time, repeat count of 2 and normalized collision energy of 35% was used for the fragmentation. Scans were obtained for the *m*/*z* range of 400–1800 Da at 50,000 resolution.

### 1.4 Protein identification and data validation

Peak lists for MS/MS spectra were extracted using the Analyst QS script MASCOT.dll, after discarding all spectra with less than ten peaks. Peptide sequences were then assigned by using MASCOT (Matrix Science, London, UK) to search the NCBI database with the following search parameters. Raw MS/MS data were searched against NCBI *Viridiplantae* entries, a total of 278115 sequences that were last updated on Dec. 31th 2011 (NIH, Bethesda, MD, USA), using the MASCOT algorithm version 2.3.02 (Matrix Science, London, UK). The MS and MS/MS mass tolerances were 3.0 Da and 1.0 Da, respectively, and up to two missed cleavages were permitted for fully tryptic peptides. Carboxamidomethyl cysteine and oxidized methionine were set as fixed and variable modifications, respectively. The false discovery rate (FDR) was determined by using a target-decoy search strategy [3]. PepDistiller [4], which facilitates the sensitive and accurate validation of MASCOT search results, was used to validate MS/MS-based peptides and the peptide FDR was controlled at 1.0%. A spectral count (SC) based, label-free quantification was implemented using the SILVER tool developed at the Beijing Proteome Research Center (BPRC) [5]. The proteins extracted from strawberries harvested before storage (initial) were used as the denominator against which the ratios of normalized spectral counts from the post-storage strawberry proteomes were compared, in order to assess any differential regulation of proteins in response to the treatments (Supplemental Table 2).
