# Supplementary material for: Data in support of comparative analysis of strawberry proteome in response to controlled atmosphere and low temperature storage using a label-free quantification
Source: Data Brief. 2015 Mar 20;3:185–8. doi: 10.1016/j.dib.2015.02.023 (PMC4510141; doi:10.1016/j.dib.2015.02.023)
Supplement: Supplementary file 1 — Supplementary Material [file mmc1.doc]

**Supplemental Table 1** – Odor description and concentration of main volatile compounds after 9 days of storage in CA, RT and LT.

| Peak Name | Odor description | Retention time (min) | CA (μg g^–1^) | RT (μg g^–1^) | LT (μg g^–1^) |
| --- | --- | --- | --- | --- | --- |
| **Esters** |  |  |  |  |  |
| Ethyl propionate | pineapple-like | 4.036 | 0.0 | 0.0 | 2.8 |
| 3-Hydroxy ethyl hexanoate | Fatty, fruity | 6.351 | 75.1 | 25.9 | 32.3 |
| Ethyl-2-butenoate | Fruity, sweet, pineapple | 7.581 | 10.1 | 0.4 | 2.5 |
| Ethyl-2-methylbutyrate | Apple-like | 7.962 | 5.9 | 0.6 | 4.6 |
| Ethyl pentanoate | Fruity, Apple-like | 9.870 | 1.0 | 1.5 | 1.1 |
| Dimethyl 3-methylglutaconate | Fruity | 10.780 | 8.2 | 52.3 | 0.9 |
| *(E)*-Ethyl-2-methyl-2-butenoate | fruity | 11.360 | 2.7 | 0.0 | 0.0 |
| Ethyl hexanoate | fruity, sweet, pineapple | 13.708 | 498.9 | 255.1 | 83.0 |
| Ethyl 3-hexenoate | pear, apple, pineapple | 14.030 | 0.0 | 0.0 | 1.0 |
| Hexyl acetate | Apple, cherry, pear, floral, sweetish | 14.236 | 0.0 | 0.0 | 1.4 |
| Phenylmethylacetate | Honey-like | 20.087 | 9.5 | 25.0 | 9.2 |
| Ethyl benzoate | sweet, fruity, cherry, grape | 20.386 | 2.9 | 1.1 | 1.8 |
| Ethyl octanoate | Pleasant, fruity, floral, wine-apricot | 21.231 | 19.4 | 14.6 | 6.4 |
| Ethyl 3-hydroxy-4-methyl-pentanoate | Fruity, berry-like | 25.937 | 2.3 | 0.7 | 1.4 |
| Ethyl cinnamate | Fruity, balsamic | 30.479 | 36.1 | 8.2 | 66.9 |
| Ethyl dodecanoate | Floral, fruity | 33.997 | 2.5 | 0.6 | 1.3 |
| Ethyl hexadecanoate | Fatty, pleasant, sweet | 40.722 | 0.0 | 0.0 | 0.8 |
| **Acids** |  |  |  |  |  |
| 2-Methyl pentanoic acid | Caramel-like | 4.308 | 0.0 | 0.0 | 0.0 |
| Trans-2-hexenoic acid | acid | 15.507 | 13.6 | 4.4 | 4.3 |
| Octanoic acid | Acid, pungent | 20.502 | 0.0 | 0.0 | 0.0 |
| **Alcohols** |  |  |  |  |  |
| 2-Methylcyclopentanol | Strawberry, coconut oil-like | 6.385 | 0.0 | 0.0 | 0.0 |
| Cis-*á,á*-5-(1-hydroxy-1-methylethyl)-2-methyl-2-vinyltetrahydrofuranmethanol | Floral | 17.173 | 7.4 | 4.3 | 3.7 |
| Linalool, 3,7-dimethyl-1,6-octadien-3-ol | Citrus-like | 17.695 | 128.0 | 66.1 | 69.4 |
| Cis-3-nonylene-1-ol | Floral | 17.864 | 5.15 | 2.5 | 2.4 |
| 2-(4-Methyl-3-cyclohexen-yl)-2-propanol | pleasant | 21.321 | 25.0 | 18.6 | 7.2 |
| 1,10-Decanediol | aromatic | 21.634 | 2.7 | 1.9 | 1.5 |
| 3,7-Dimethyl-2,6-octadien-1-ol | Floral, sweet | 23.231 | 2.8 | 2.5 | 0.3 |
| 3-Methyl-benzyl alcohol | Pleasant, aromatic | 23.670 | 0.0 | 0.0 | 1.1 |
| 3,7,11-Trimethyl-1,6,10-dodecatrien-3-ol | Floral | 33.232 | 290.3 | 253.0 | 240.1 |
| **Terpenes** |  |  |  |  |  |
| 1-Methyl-4-(1-methylethenyl)-cyclohexene | terpenic | 14.894 | 2.9 | 2.3 | 2.4 |
| 3,7,11-Trimethyl-1,3,6,10-dodecatetraene | Green apple | 31.555 | 3.0 | 1.5 | 1.6 |
| 1-Caryophyllene | spicy | 28.943 | 5.9 | 2.4 | 8.1 |
| Cis-*â*-farnesene | Green apple | 29.859 | 4.6 | 1.3 | 2.8 |
| **Furanones** |  |  |  |  |  |
| 4-Methoxy-2,5-dimethyl-3(2H)-furanone (DMMF) | Sweet, Caramel-like | 16.029 | 458.3 | 469.8 | 85.7 |
| 5-Hexyldihydro-2(3H)-furanone (*ã*-dodecalactone) | Coconut milk-like | 35.924 | 98.7 | 53.6 | 20.4 |
| **Others** |  |  |  |  |  |
| Chamomile blue | Apple-like | 20.986 | 5.2 | 0.0 | 0.0 |
| Eugenol | spicy | 26.778 | 7.2 | 5.0 | 1.9 |
| Trans-2-hexenal | grassy, pungent | 8.172 | 39.8 | 17.3 | 37.1 |
