# Supplementary material for: Data in support of comparative analysis of strawberry proteome in response to controlled atmosphere and low temperature storage using a label-free quantification
Source: Data Brief. 2015 Mar 20;3:185–8. doi: 10.1016/j.dib.2015.02.023 (PMC4510141; doi:10.1016/j.dib.2015.02.023)
Supplement: Supplementary file 2 — Supplementary Material [file mmc2.doc]

**Supplemental Table 2** – Differentially expressed proteins of the strawberry proteome, in the three clusters, after 9 days of storage in CA, RT and LT.

| No.^a^ | Protein name | Organism | Accession number ^b^ | | Mass/pI ^c^ | | Mascot score ^d^ | | | Matched peptides number | | Sequence coverage (%) | | Protein relative abundance ^e^ | | | |
| --- | --- | --- | --- | --- | --- | --- | --- | --- | --- | --- | --- | --- | --- | --- | --- | --- | --- |
| **Cluster 1** | |  |  |  | |  | | |  | | | |  |  | | | |
| 1 | transcription factor BTF3-like isoform 1 | *Fragaria vesca* | gi|470126975 | | 18.30/5.79 | | 631 | | | 2 | | 5 | |  | | | |
| 2 | vicilin-like antimicrobial peptides 2-1-like | *Fragaria vesca* | gi|470112718 | | 58.50/6.93 | | 417 | | | 3 | | 16 | | 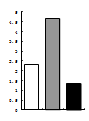 | | | |
| 3 | quinone oxidoreductase-like protein At1g23740, chloroplastic-like | *Fragaria vesca* | gi|470137735 | | 34.19/5.72 | | 83 | | | 2 | | 10 | | 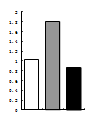 | | | |
| 4 | malate dehydrogenase, mitochondrial-like | *Fragaria vesca* | gi|470115832 | | 35.62/8.46 | | 273 | | | 3 | | 12 | | 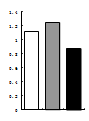 | | | |
| 5 | dihydrolipoyl dehydrogenase 1, mitochondrial-like | *Fragaria vesca* | gi|470117795 | | 53.98/6.93 | | 530 | | | 4 | | 18 | | 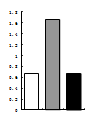 | | | |
| 6 | uncharacterized protein LOC101306848 isoform 3 | *Fragaria vesca* | gi|470101465 | | 34.80/4.72 | | 95 | | | 6 | | 45 | | 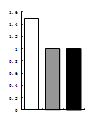 | | | |
| 7 | probable protein disulfide-isomerase A6-like | *Fragaria vesca* | gi|470132774 | | 39.67/6.11 | | 246 | | | 3 | | 17 | | 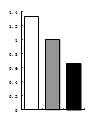 | | | |
| 8 | 12S seed storage protein CRU2-like | *Fragaria vesca* | gi|470127983 | | 35.82/5.25 | | 255 | | | 2 | | 13 | | 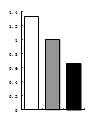 | | | |
| 9 | luminal-binding protein 5-like | *Fragaria vesca* | gi|470129154 | | 73.66/5.13 | | 56 | | | 1 | | 7 | | 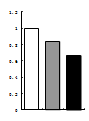 | | | |
| 10 | pyruvate kinase, cytosolic isozyme-like | *Fragaria vesca* | gi|470134411 | | 57.51/6.30 | | 291 | | | 5 | | 24 | | 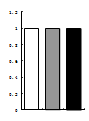 | | | |
| 11 | malate dehydrogenase, cytoplasmic-like | *Fragaria vesca* | gi|470120564 | | 35.60/6.01 | | 192 | | | 10 | | 28 | | 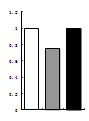 | | | |
| 12 | L-ascorbate peroxidase, cytosolic-like | *Fragaria vesca* | gi|470117066 | | 27.28/5.68 | | 160 | | | 4 | | 19 | | 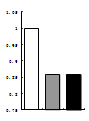 | | | |
| 13 | succinyl-CoA ligase [ADP-forming] subunit alpha-1, mitochondrial-like | *Fragaria vesca* | gi|470126989 | | 35.03/9.06 | | 251 | | | 2 | | 31 | | 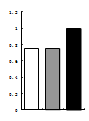 | | | |
| 14 | uncharacterized protein LOC101304165 | *Fragaria vesca* | gi|470113601 | | 57.00/5.96 | | 138 | | | 9 | | 54 | | 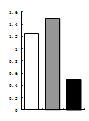 | | | |
| 15 | leucoanthocyanidin dioxygenase-like | *Fragaria vesca* | gi|470125461 | | 42.86/5.48 | | 590 | | | 3 | | 34 | | 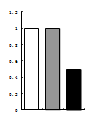 | | | |
| 16 | major allergen Pru av 1-like | *Fragaria vesca* | gi|470121687 | | 17.44/5.39 | | 72 | | | 2 | | 12 | | 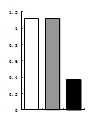 | | | |
| 17 | uncharacterized protein LOC101314953 | *Fragaria vesca* | gi|470128841 | | 40.70/6.92 | | 481 | | | 12 | | 77 | | 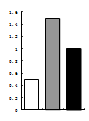 | | | |
| 18 | leucine aminopeptidase 3, chloroplastic-like | *Fragaria vesca* | gi|470101341 | | 59.98/7.55 | | 189 | | | 7 | | 25 | | 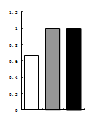 | | | |
| 19 | 12S seed storage protein CRU1-like | *Fragaria vesca* | gi|470107217 | | 64.49/6.77 | | 102 | | | 1 | | 9 | | 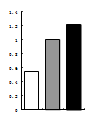 | | | |
| 20 | protein disulfide-isomerase-like | *Fragaria vesca* | gi|470109183 | | 55.86/4.94 | | 90 | | | 3 | | 18 | | 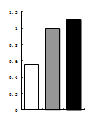 | | | |
| 21 | uncharacterized protein LOC101299909 isoform 1 | *Fragaria vesca* | gi|470106646 | | 17.17/6.33 | | 43 | | | 8 | | 26 | | 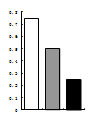 | | | |
| 22 | 40S ribosomal protein S20-2-like | *Fragaria vesca* | gi|470145076 | | 13.64/9.51 | | 644 | | | 2 | | 15 | | 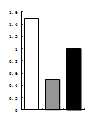 | | | |
| **Cluster 2** | |  |  |  | |  | |  | | |  | | | |  |  | |
| 1 | probable fructose-bisphosphate aldolase 3, chloroplastic-like | *Fragaria vesca* | gi|470126093 | | 42.65/8.56 | | 477 | | | 5 | | 22 | | 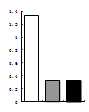 | | | |
| 2 | heat shock protein STI-like | *Fragaria vesca* | gi|470125359 | | 65.66/6.02 | | 416 | | | 4 | | 30 | | 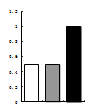 | | | |
| 3 | V-type proton ATPase subunit E-like | *Fragaria vesca* | gi|470125749 | | 26.46/8.29 | | 126 | | | 1 | | 18 | | 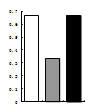 | | | |
| 4 | uncharacterized protein LOC101312443 | *Fragaria vesca* | gi|470126658 | | 24.59/4.96 | | 359 | | | 2 | | 55 | | 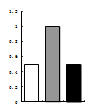 | | | |
| 5 | importin subunit alpha-1-like | *Fragaria vesca* | gi|470117953 | | 58.10/5.25 | | 553 | | | 4 | | 42 | | 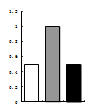 | | | |
| 6 | non-functional NADPH-dependent codeinone reductase 2-like | *Fragaria vesca* | gi|470126493 | | 36.14/5.93 | | 32 | | | 8 | | 71 | | 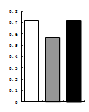 | | | |
| 7 | adenylate kinase B-like | *Fragaria vesca* | gi|470123087 | | 26.50/8.54 | | 308 | | | 6 | | 37 | | 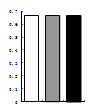 | | | |
| 8 | triosephosphate isomerase, cytosolic-like | *Fragaria vesca* | gi|470143704 | | 27.13/6.34 | | 93 | | | 5 | | 29 | | 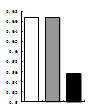 | | | |
| 9 | legumin B-like | *Fragaria vesca* | gi|470115872 | | 56.53/6.32 | | 120 | | | 1 | | 15 | | 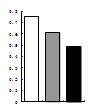 | | | |
| 10 | legumin A-like | *Fragaria vesca* | gi|470107215 | | 56.61/6.84 | | 74 | | | 1 | | 16 | | 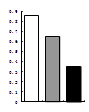 | | | |
| 11 | caffeic acid 3-O-methyltransferase-like | *Fragaria vesca* | gi|470144514 | | 39.80/5.31 | | 44 | | | 3 | | 47 | | 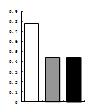 | | | |
| 12 | phosphoglycerate kinase, cytosolic-like | *Fragaria vesca* | gi|470141725 | | 42.29/6.20 | | 107 | | | 11 | | 54 | | 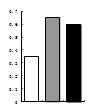 | | | |
| 13 | peptidyl-prolyl cis-trans isomerase 1-like | *Fragaria vesca* | gi|470107009 | | 18.27/8.53 | | 180 | | | 4 | | 71 | | 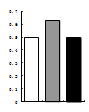 | | | |
| 14 | fructose-bisphosphate aldolase cytoplasmic isozyme-like | *Fragaria vesca* | gi|470134361 | | 38.53/7.59 | | 102 | | | 2 | | 34 | | 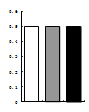 | | | |
| 15 | probable nucleoredoxin 1-like | *Fragaria vesca* | gi|470122914 | | 65.45/4.84 | | 360 | | | 1 | | 16 | | 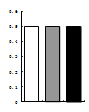 | | | |
| 16 | nucleoside diphosphate kinase 1-like | *Fragaria vesca* | gi|470142572 | | 16.35/6.84 | | 65 | | | 2 | | 25 | | 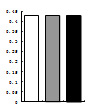 | | | |
| 17 | cysteine proteinase inhibitor 12-like isoform 1 | *Fragaria vesca* | gi|470143070 | | 26.29/6.17 | | 89 | | | 3 | | 31 | | 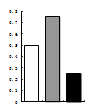 | | | |
| 18 | uncharacterized protein LOC101310704 | *Fragaria vesca* | gi|470126646 | | 14.44/9.19 | | 658 | | | 5 | | 43 | | 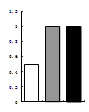 | | | |
| 19 | glycinin G4-like | *Fragaria vesca* | gi|470107221 | | 57.89/8.08 | | 126 | | | 2 | | 17 | | 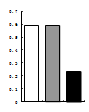 | | | |
| 20 | pyruvate decarboxylase isozyme 2-like | *Fragaria vesca* | gi|470133355 | | 65.11/5.91 | | 231 | | | 4 | | 53 | | 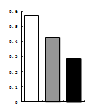 | | | |
| 21 | nascent polypeptide-associated complex subunit alpha-like protein-like | *Fragaria vesca* | gi|470141512 | | 21.93/4.30 | | 137 | | | 2 | | 24 | | 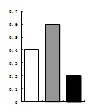 | | | |
| 22 | probable mediator of RNA polymerase II transcription subunit 37e-like | *Fragaria vesca* | gi|470133735 | | 70.92/5.11 | | 36 | | | 1 | | 6 | | 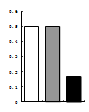 | | | |
| 23 | naringenin,2-oxoglutarate 3-dioxygenase-like | *Fragaria vesca* | gi|470102750 | | 41.08/5.51 | | 111 | | | 2 | | 13 | | 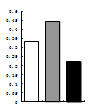 | | | |
| 24 | translationally-controlled tumor protein homolog | *Fragaria vesca* | gi|470121591 | | 18.91/4.41 | | 85 | | | 2 | | 25 | | 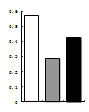 | | | |
| 25 | NADP-dependent malic enzyme-like | *Fragaria vesca* | gi|470102042 | | 65.07/6.32 | | 115 | | | 7 | | 68 | | 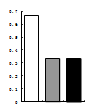 | | | |
| 26 | proteasome subunit beta type-1-like | *Fragaria vesca* | gi|470142441 | | 24.38/6.95 | | 556 | | | 4 | | 34 | | 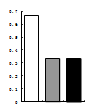 | | | |
| 27 | ankyrin repeat domain-containing protein 2-like | *Fragaria vesca* | gi|470107539 | | 38.90/4.44 | | 60 | | | 2 | | 21 | | 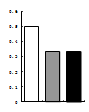 | | | |
| 28 | superoxide dismutase [Cu-Zn]-like | *Fragaria vesca* | gi|470103627 | | 15.24/5.76 | | 66 | | | 3 | | 16 | | 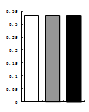 | | | |
| **Cluster 3** | |  |  |  | |  | |  | | |  | | | |  | |  |
| 1 | 26S protease regulatory subunit 6B homolog | *Fragaria vesca* | gi|470142843 | | 46.03/5.48 | | 79 | | | 2 | | 18 | | 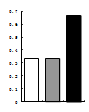 | | | |
| 2 | ruBisCO large subunit-binding protein subunit beta, chloroplastic-like | *Fragaria vesca* | gi|470117611 | | 63.75/5.92 | | 52 | | | 6 | | 56 | | 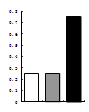 | | | |
| 3 | uncharacterized protein LOC101294513 | *Fragaria vesca* | gi|470144911 | | 75.22/5.46 | | 14 | | | 3 | | 24 | | 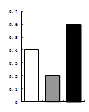 | | | |
| 4 | uncharacterized protein LOC101292566 | *Fragaria vesca* | gi|470109570 | | 10.81/4.80 | | 131 | | | 2 | | 16 | | 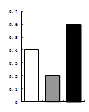 | | | |
| 5 | legumin A-like | *Fragaria vesca* | gi|470107219 | | 62.76/8.18 | | 88 | | | 2 | | 11 | | 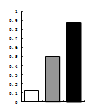 | | | |
| 6 | BAHD acyltransferase At5g47980-like | *Fragaria vesca* | gi|470136274 | | 51.33/5.57 | | 389 | | | 4 | | 39 | | 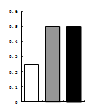 | | | |
| 7 | calmodulin-related protein-like | *Fragaria vesca* | gi|470104452 | | 16.85/4.11 | | 279 | | | 1 | | 4 | | 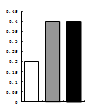 | | | |
| 8 | tubulin beta-1 chain-like | *Fragaria vesca* | gi|470116123 | | 50.25/4.74 | | 62 | | | 4 | | 27 | | 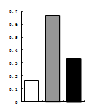 | | | |
| 9 | 5-methyltetrahydropteroyltriglutamate--homocysteine methyltransferase-like | *Fragaria vesca* | gi|470111481 | | 84.58/6.52 | | 124 | | | 2 | | 22 | | 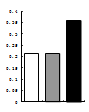 | | | |
| 10 | 60S acidic ribosomal protein P2B-like | *Fragaria vesca* | gi|470134336 | | 11.45/4.56 | | 47 | | | 5 | | 41 | | 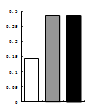 | | | |
| 11 | cold shock domain-containing protein 4-like | *Fragaria vesca* | gi|470107029 | | 19.18/5.92 | | 135 | | | 1 | | 14 | | 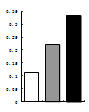 | | | |
| 12 | ubiquitin-NEDD8-like protein RUB2-like | *Fragaria vesca* | gi|470115767 | | 17.20/5.78 | | 16 | | | 3 | | 35 | | 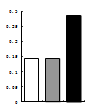 | | | |
| 13 | UTP--glucose-1-phosphate uridylyltransferase-like isoform 1 | *Fragaria vesca* | gi|470104393 | | 51.88/6.29 | | 57 | | | 5 | | 24 | | 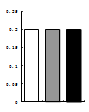 | | | |
| 14 | LOW QUALITY PROTEIN: dehydrin COR47-like | *Fragaria vesca* | gi|470109984 | | 28.56/5.37 | | 144 | | | 2 | | 6 | | 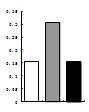 | | | |
| 15 | glyceraldehyde-3-phosphate dehydrogenase-like | *Fragaria vesca* | gi|470149016 | | 36.39/8.24 | | 221 | | | 8 | | 36 | | 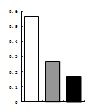 | | | |
| 16 | elongation factor 1-alpha 1-like | *Fragaria vesca* | gi|470148685 | | 49.38/9.15 | | 148 | | | 1 | | 11 | | 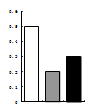 | | | |
| 17 | peroxiredoxin-2B-like | *Fragaria vesca* | gi|470128097 | | 17.48/5.34 | | 12 | | | 5 | | 21 | | 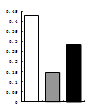 | | | |
| 18 | elongation factor 2-like | *Fragaria vesca* | gi|470115786 | | 94.18/5.83 | | 133 | | | 2 | | 7 | | 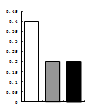 | | | |
| 19 | 14-3-3-like protein-like | *Fragaria vesca* | gi|470136735 | | 29.71/4.75 | | 263 | | | 1 | | 5 | | 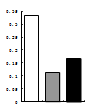 | | | |
| 20 | cell division cycle protein 48 homolog | *Fragaria vesca* | gi|470137394 | | 89.52/5.07 | | 292 | | | 2 | | 18 | | 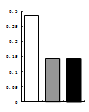 | | | |
| 21 | copper transport protein ATOX1-like, partial | *Fragaria vesca* | gi|470145863 | | 11.45/6.92 | | 80 | | | 1 | | 7 | | 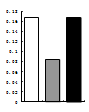 | | | |
| 22 | chalcone--flavonone isomerase-like | *Fragaria vesca* | gi|470144168 | | 23.51/4.85 | | 78 | | | 3 | | 18 | | 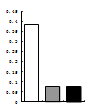 | | | |
| 23 | enolase-like | *Fragaria vesca* | gi|470134388 | | 47.94/5.76 | | 134 | | | 7 | | 41 | | 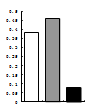 | | | |

^a^ Protein no. and order correspond to the hierarchical clustering analysis in Fig. 5.

^b^ Accession numbers according to NCBI *Viridiplantae* database.

^c^ Experimental mass (kDa) and isoelectric point (pI) of identified proteins.

^d^ Mascot score reported after searching against the NCBI *Viridiplantae* database.

^e^ Relative abundances of normalized protein expression; values are expressed as the mean of three replications. White, grey and black bars show CA, RT and LT storage, respectively.
